# Survey among ESGE members on leiomyosarcoma morcellation incidence

**DOI:** 10.1186/s10397-017-1027-z

**Published:** 2017-12-04

**Authors:** Vasilios Tanos, Hans Brölmann, Rudi Leon DeWilde, Peter O’Donovan, Elina Symeonidou, Rudi Campo

**Affiliations:** 1 St. Georges Medical School, Nicosia University, Nicosia, Cyprus; 2 Department of Obstetrics and Gynaecology, Aretaeio Hospital, Nicosia, Cyprus; 3 Department of Obstetrics and Gynaecology, VU University Medical Centre, De Boelelaan 1117, 1181HV Amsterdam, The Netherlands; 4 Clinic of Gynaecology, Obstetrics and Gynaecological Oncology, University Hospital for Gynaecology, Pius-Hospital Oldenburg, Medical Campus University of Oldenburg, Oldenburg, Germany; 5 Obstetrics and Gynaecological Oncology Yorkshire Clinic, Bradford Road, Bingley, West Yorkshire BD16 1TW UK; 6 Nicosia, Cyprus; 7 European Society Gynaecological Endoscopy, European Academy for Gynaecological Surgery, LIFE, Tiensevest, 168, 3000 Leuven Leuven, Belgium

**Keywords:** Leiomyoma uteri, Leiomyosarcoma, Leiomyosarcoma incidence, Laparoscopy, Morcellation, Power morcellation complication

## Abstract

**Background:**

Increased awareness of leiomyosarcoma (LMS) risk during myomectomy or hysterectomy is essential. Objective and correct reasoning should prevail on any decision regarding the extent and type of surgery to employ. The anticipated risk of a sarcoma after myoma or uterus morcellation is low, and the frequency of leiomyosarcoma especially in women below the age of 40 is very rare. The prevalence data has a wide range and is therefore not reliable. The European Society of Gynaecological Endoscopy (ESGE) initiated a survey among its members looking into the frequency of morcellated leiomyosarcoma after endoscopic surgery.

The ESGE Central office sent 3422 members a structured electronic questionnaire with multiple answer choices for each question. After 3 months, the answers were classified with a unique number in the EXCEL spread sheet. Statistical analysis was done using the SPSS v.18.

**Results:**

Out of 3422 members, 294 (8.6%) gynaecologists replied to the questionnaire; however, only 240 perform myomectomies by laparoscopy and hysteroscopy and hysterectomies by laparoscopy. The reported experience in performing laparoscopic myomectomy, hysteroscopic myomectomy, laparoscopic hysterectomy (LH), and laparoscopic subtotal hysterectomy (LSH) on an average was 10.8 (1–32) years. The vast majority of 67.1% had over 5 years of practice in laparoscopic surgery. The total number of 221 leiomyosarcoma was reported among 429,777 minimally invasive surgeries (laparoscopic and hysteroscopic myomectomies and LH and LSH), performed by all doctors in their lifetime. The overall reported sarcoma risk of all types of endoscopic myoma surgeries has been estimated to be 1.5% of operations which is very rare. Categorizing by type, 57 (0.06%) LMS were operated by laparoscopic myomectomy and 54 (0.07%) by hysteroscopic myomectomy, while 38 (0.13%) leiomyosarcoma operated by laparoscopic subtotal hysterectomy and 72 (0.31%) by laparoscopic hysterectomy. The probability of a sarcoma after morcellation to be falsely diagnosed by histopathology as a benign tumour and later identified as a sarcoma in a later examination has been reported and calculated to be 0.2%. The low risk of a sarcoma is also reflected by the small number of surgeries, where only 32 doctors reported that they operated once, 29 twice, and 18 operated on 3–10 sarcomas by laparoscopy during their lifetime.

**Conclusion:**

The survey demonstrated that myomectomy by hysteroscopy or laparoscopy has similar risks of sarcoma with an estimated incidence of 0.07%, much lower than that by laparoscopic hysterectomy and subtotal hysterectomy. Hence, for young patients with myoma infertility problem and low risk for LMS, myomectomy by MIS can be the first option of treatment. The fact that only 12.5% (216/1728) of uterine sarcoma cases are operated laparoscopically demonstrates the surgeons’ awareness and alertness about LMS and the potential of spreading sarcomatous cells after myoma/uterus power morcellation.

## Background

Morcellation has been used for a long time during minimally invasive surgery (MIS) to extract pathological tissue from the abdominal cavity through a trocar or via the vagina. The FDA did a meta-analysis of the data from 18 studies and reported a risk of a uterine sarcoma in patients with presumed fibroids to be 0.28% [1]. It was then advised to avoid morcellation of uterine myomas suspicious for sarcoma in order to prevent the spread of sarcomatous cells and upstaging of the cancer status of the patient. The professional community and representatives of many scientific societies published their opinions on the matter [2–7], however, were unable to unanimously recommend for or against laparoscopic myomectomy or hysterectomy due to lack of solid scientific data. The dilemma of whether to counsel patients with fibroids to choose laparoscopic surgery with its established benefits or laparotomy to escape the small risks related to fibroid morcellation is still an open discussion [8].

Uterine fibroids are a common disorder with an estimated incidence of 20–40% in women during their reproductive years [9, 10]. In contrast, leiomyosarcoma (LMS) of the uterus is a rare entity with an annual incidence quoted between 0.014–0.28% [1, 11, 12]. Excluding the carcinosarcoma or Mixed Müllerian Tumour (MMT), LMS accounts for 70% and stromal sarcoma for 30% of all uterine sarcomas [13]. The true prevalence of uterine sarcoma in presumed fibroids is not known, and meta-analyses based on retrospective trials have shown a wide range of prevalence (0.014–0.45%). Age and certain imaging characteristics such as ‘lacunes’ suggesting necrosis and increased central vascularisation of the tumour are associated with a relatively higher risk of uterine sarcoma, although the overall risk remains low. There is not enough evidence to estimate this risk in individual patients [8]. Uterine sarcomas represent 2–7% of all uterine malignancies. Reliable figures for the incidence of smooth muscle tumours of unknown malignant potential (STUMP) and cellular fibroids are poorly documented. A LMS may spread locally, regionally, or by haematogenous dissemination. Local and regional spread may result in an abdominal or pelvic tumour causing gastrointestinal and/or urinary tract symptoms. Haematogenous dissemination most often spreads to the lungs. LMS typically appears around a median age of 50–55 years and is highly malignant, and in most cases, recurrences are detected within 2 years. A major prognostic factor is the extent of tumour spread [13]. Five-year survival ranges from 17 to 55%. Survival of patients with a LMS is strongly associated with the number of mitoses per 10 high power fields (× 100 magnification): 1–4, 98%; 5–9, 42%; ≥10; 15%. A LMS embedded and confined to the uterus that is removed ‘end bloc’ is associated with a better survival rate of up to 83% [14, 15].

Increased awareness of sarcoma risk is essential, but objective and correct reasoning should predominate any decision regarding the extent and type of surgery selected. The currently available data on prevalence of a LMS when operating on a presumed leiomyoma is imprecise and unreliable. Recently, Pritts et al. analysed 67 prospective studies in which 5951 women underwent surgery for fibroids and only two were found to have LMS (1 in 2975 or 0.03%). When they analysed an additional 66 retrospective studies, out of 23,926 women having surgery for fibroids, 22 were found to have LMS (1 in 1087 or 0.09%) [15].

The European Society of Gynaecological Endoscopy (ESGE), in an effort to be as objective as possible, initiated a survey among its members to collect data from each centre and surgeon individually. The survey results have assisted in the evaluation of sarcoma frequency after myoma morcellation in absolute numbers, reflecting European surgeons’ current endoscopic surgery practices.

## Methods

The ESGE central office sent its 3422 members a structured electronic questionnaire with multiple answer choices in July, 2014. Free text options and comments were also available for some of the questions. A letter accompanied the questionnaire (see Table 1) explaining the reason for the survey and read as follows: “Recently case reports on fibroid morcellation disseminating unexpected malignancy attracted the attention of gynaecologists but also public attention mainly through various websites. ESGE, in an effort to gain more information on that very rare neoplasia but also critical issue, would like to run a survey among its members in order to be able to give more information and advice to gynaecologists performing laparoscopic surgery. The doctors were asked to answer the questions, taking into consideration their management on symptomatic fibroids and/or enlarged uterus before the press release from the FDA.”

**Table 1:** Descriptive statistics according to survey questions

Questions	Number of answers	Mean	Mode	Standard deviation	Sum	Pearson’s correlation coefficient	
Q1: How many years have you been practicing laparoscopic myomectomy and hysterectomy?	240	10.78	5	7.265	2587		
Q2: How many uterine sarcomas have received a laparoscopic surgical approach?	280	.77	0	2.023	216	Q3	.427
						Q5	.451
						Q9	.461
Q3: How many uterine sarcomas have you seen in your lifetime practice?	280	6.17	2	9.008	1728	Q2	.427
Q4: How many laparoscopic myomectomies do you perform annually?	238	29.08	0	47.632	6920	Q5	.412
Q5: How many sarcomas did you encounter until today after laparoscopic myomectomies?	236	.24	0	.916	57	Q2	*.451*
						Q4	.412
						Q9	*.492*
Q6: How many laparoscopic subtotal hysterectomies you do per year?	237	28.81	0	52.492	6828	Q7	.484
Q7: How many sarcomas did you have until today after laparoscopic subtotal hysterectomies?	236	.16	0	.470	38	Q6	.484
Q8: How many laparoscopic hysterectomies you do per year?	237	43.90	30	49.018	10.404		
Q9: How many sarcomas did you have until today after laparoscopic hysterectomies?	235	.31	0	1.191	72	Q2	.461
						Q5	.492
Q10: How many hysteroscopic myomectomies do you perform annually?	233	31.10	20	29.233	7246		
Q11: How many sarcomas did you encounter until today after hysteroscopic myomectomies?	234	.23	0	1.087	54		
Q12: Myomectomy cases reported as leiomyoma and review of the slides revealed a sarcoma	233	.19	0	.556	44		

Using the ESGE server and website, in conjunction with the “Survey Monkey” programme, the central office sent the questionnaire out electronically. By mid-September 2014, the survey was closed and the doctors who responded were automatically identified in an EXCEL spread sheet. E-mail addresses were used for the identification of each individual, while a serial number was also used to separate and establish the study group, thereby avoiding mistakes and enabling anonymous statistical analysis.

The probability of LMS was based on the average of individual results from each gynaecologist. The probability of a surgeon identifying a sarcoma while performing MIS (any type of surgery) has been estimated individually based on the total number of sarcomas divided by the total number of surgeries performed in a lifetime (number of surgeries performed annually multiplied by the years of practice). The overall probability is the average of each individual probability of sarcoma, presenting the mean of the means of each individual.

### Statistical analysis

The statistical analysis was performed by using SPSS v.18 (Statistical Package for Social Sciences). The first part of the questionnaire consisted of 12 open-ended questions. Descriptive statistics (mean, mode, standard deviation, and sum) and frequency charts (bar charts) are used in order to give a screenshot of the sampled data. In order to present the data in bar charts, the quantitative data have been categorized in an ordinal form. Correlations, calculated with Pearson’s correlation coefficient, are used as raw data (not categorized) in order to find the interaction between the answers. Pearson’s correlation coefficient takes values between − 1 and 1, where − 1 proves strong negative correlation between the answers, zero value (0) shows no correlation and 1 proves strong positive correlation (Table 1). Where correlation coefficient exceeds or is close to |1|, the correlation is considered to be strong, and where the significance value (*p* value) is less than 0.01 is considered to be statistically significant. Correlations are also visualized through clustered bar charts. Based on the sampled data, the probability of fact occurrence is also calculated.

## Results

Out of 3422 ESGE members who received the questionnaire, 294 responded (8.6%) to the survey call. A total of 280 (95.2%) members were included in the final statistical analysis. Forty doctors received a second invitation because of incomplete survey answer. At the end, 14 respondents could not be included. Among 280 responders, 240 reported experience performing laparoscopic hysterectomies, myomectomies, and hysteroscopic myoma resections. The reported average experience in MIS was 10.7 years while the vast majority 67.1% had over 5 years of practice in laparoscopic surgery.

Survey results show that out of 1728 lifetime LMS seen by surgeons participating in this survey, 216 cases (12.5%) received laparoscopic surgery. The sum of the diagnosed sarcomas after each individual type of surgery in a lifetime as shown in Table 1 (sum (Q5 + Q7 + Q9 + Q11) = 221) which is verified by the sum of the general questions of the total number of uterine sarcomas calculated and seen in a lifetime (Table 1: sum Q2 = 216) strengthens the reliability of the answers given by the participants. The overall probability of finding a sarcoma after endoscopic surgery (myomectomy or hysterectomy) is 1.5%. The lowest probability of sarcoma is after laparoscopic myomectomy 0.6% and after hysteroscopic myomectomy 0.7% while the greatest probability of sarcoma is after laparoscopic hysterectomy 3.1%. The probability of sarcoma after laparoscopic subtotal hysterectomy is close to the overall probability and is equal to 1.3%. The number of doctors who participated in this survey as well as the high number of operations reported demonstrates the group’s large exposure to gynaecological endoscopic surgery and strongly supports the reliability of these results.

### Experience in MIS

**Fig. 1:**
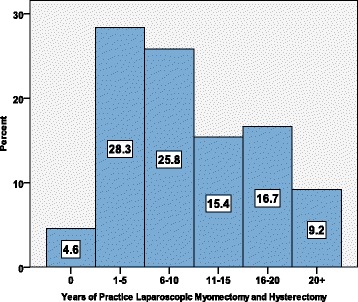
The exposure of doctors to laparoscopic surgery. The percentage of gynae-surgeons practicing laparoscopic myomectomy and hysterectomy, according to years’ interval

**Fig. 2:**
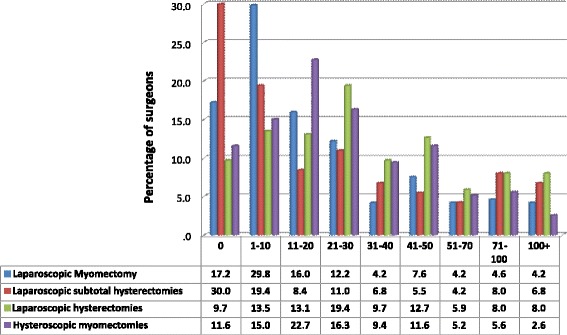
Exposure to endoscopic surgeries on an annual basis

The vast majority of responders (67.1%) reported over 5 years of practice in laparoscopic surgery, performing between 29 and 44 operations of each type (LH, LSH, LM, HM) of MIS on an annual basis. The overall exposure of doctors to laparoscopic surgery is demonstrated in Fig. 1. The rate of gynaecologists practicing laparoscopic myomectomy and hysterectomy according to number of years’ (time interval) shows that majority of these surgeons (54.1%) have 1 to 10 years of experience and 32.3% have 11 to 20 years of experience in MIS. Only 4.6% of the doctors participated in our survey reported less than a year of experience while 9.2% of the most experienced surgeons reported more than 20 years of laparoscopic surgery experience. The experience of the surgeons according to the type and number of operations performed annually is demonstrated in Fig. 2. The first set of bars presents an average of 17% of doctors that do not perform the described endoscopic surgeries; however, they still reported the number of sarcomas found during their lifetime. 49.3% reported that they perform 1–30 MIS and another 33.7% more than 30 MIS per year. The remaining 30% of the doctors reported that they do not perform LSH at all. Majority of doctors reported that they perform 30 laparoscopic hysterectomies and 20 hysteroscopic myomectomies on an annual basis. Statistical analysis revealed that the average number of annual LM is 29, LSH is 28, LH is 44, and HM is 31.

The most frequent type of endoscopic surgery performed is LH, and the least preferred surgical approach is LSH. Figure 2 demonstrates surgeons, with the least exposure to MIS (1–10 cases/annum), performed more myomectomies and subtotal hysterectomies than LH compared to more experienced surgeons with exposure to more than 11 endoscopic cases per year, on average. There was greater preference for LH than LSH by all experienced surgeons who performed more than 11 cases per year of MIS. The higher rate of hysteroscopic myomectomies, being 22.5 and 16.5%, were performed by surgeons reporting between 11–20 and 21–30 operations per year, respectively. Surgeons with exposure to MIS in more than 31 cases per year performed less hysteroscopic myomectomies, between 2.5 and 12%.

### Incidence of uterine leiomyosarcomas

The total number of sarcomas encountered by all doctors after LM is 57, after LSH is 38, after LH is 72, and after HM is 54. The majority of doctors (71%), however, stated that they have never seen a sarcoma after any of these surgery approaches. The most frequent type of endoscopic surgery performed is the LH estimated to be 10,404 operations per year as calculated by all doctors together in this survey. The least frequent surgical approach is subtotal LM, for which 6828 cases are performed on an annual basis by all respondents.

All participants as a group have seen 1728 uterine sarcomas in their lifetime while the most common answer on an average is between two and six cases per lifetime, with a large standard deviation of 9008. Out of 1728 uterine sarcomas, only 216 cases have received a laparoscopic surgical approach. There is a positive and strong correlation between the number of uterine sarcomas encountered in a lifetime with the laparoscopic surgical approach (Pearson’s *r* = 0.427), with the total number of sarcomas encountered after laparoscopic myomectomies (Pearson’s *r* = 0.451), and with the total number of laparoscopic hysterectomies (Pearson’s *r* = 0.461) which all reach statistically significant results (*p* value < 0.001) as shown in Table 1.

The total number of MIS surgeries described in Table 2 has been calculated by the years of endoscopic surgery experience multiplied by the number of doctors. The total number of LMS identified in a lifetime was based on the answer of the question 2 (sum = 216), which is validated by the sum of the questions 5, 7, 9, and 11 (sum = 221). The same exercise was calculated for each type of surgery as shown in Table 2. The overall sarcoma risk after endoscopic surgery including all types of surgeries in this group is 0.15% of operations. Analysis of Table 2 clearly demonstrates that the highest risk of sarcoma is after LH 3.1%, followed by LSH 1.3%, whereas the lowest risk appears after HM and LM being 0.7 and 0.6%, respectively.

**Table 2:** The frequency and probability of sarcoma in general and according to type of surgery as calculated by the number and types of operations performed in lifetime and the total number of sarcomas diagnosed in a lifetime by each surgeon

Type of surgery	Number of doctors	Total number surgeries by all doctors in their lifetime	Total number of sarcomas identified in their lifetime	Probability of sarcoma per 1000
All types of surgeries	218	429.777	221 ≈ 216^a^	1.5
Laparoscopic myomectomies	236	103.576	57	0.6
Laparoscopic subtotal hysterectomies	236	106.022	38	1.3
Laparoscopic hysterectomies	235	134.808	72	3.1
Hysteroscopic myomectomies	234	87.842	54	0.7

^a^The sum of the general question of the total number of uterine sarcomas calculated and seen in lifetime (Table 1: sum Q2 = 216). The sum of the diagnosed sarcomas after each individual type of surgery in lifetime as shown in Table 1: sum (Q5 + Q7 + Q9 + Q11) = 221)

**Fig. 3:**
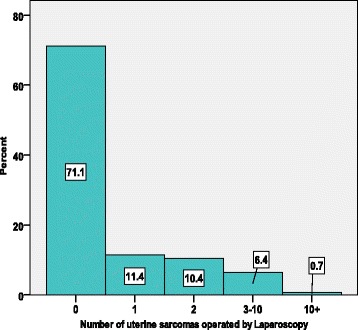
Percentage of gynae-surgeons and the number of sarcomas operated by laparoscopy

**Fig. 4:**
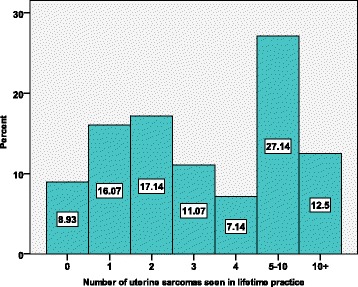
The percentage of doctors and the number of uterine sarcomas seen in their lifetime

The percentage of surgeons and the number of sarcomas operated by laparoscopy is demonstrated in Fig. 3. The vast majority of the gynaecological surgeons (71.1%) never had a LM or LH that histopathology later reported a sarcoma. Of the remaining 28.9% who did operate on sarcomas by laparoscopy during their lifetime, 11.4% experienced such an event only once, 0.4% twice, and 6.4% operated 3–10 times. The percentage of doctors and the number of uterine sarcomas seen in their lifetime is demonstrated in Fig. 4.

As demonstrated in Table 1, of those doctors who reported that they have seen sarcomas, most of them have seen a maximum of two cases in their lifetime. Once the answers are categorized, the majority of the doctors (27.14%) are grouped in the 5–10 lifetime cases, and approximately half of the doctors (51.42%) have seen 1–4 sarcomas while the remaining 12.5% have seen more than 10 cases in their lifetime.

**Fig. 5:**
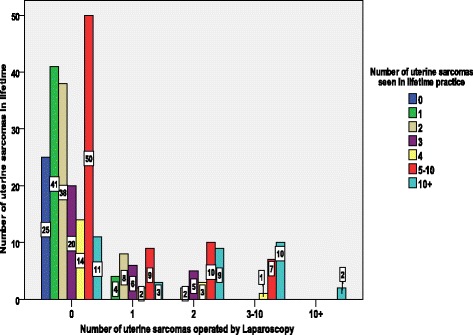
The interaction between the number of uterine sarcomas seen in lifetime and the number of uterine sarcomas treated by laparoscopy

Figure 5 demonstrates that the majority, 62% (174/280), of endoscopic surgeons are aware of the sarcoma risk, and accordingly did not operate on sarcomas by laparoscopy during their lifetime. Among the 280 gynaecologists who operated by laparoscopy, 25 have never seen a uterine sarcoma during their lifetime practice. The 174 doctors that came across a sarcoma at least once during their lifetime practice did not perform laparoscopic surgery. However, 32 doctors reported that they operated once, 29 twice, and 18 doctors operated on 3–10 sarcomas by laparoscopy and only 2 operated more than 10 sarcomas by laparoscopy.

Only 0.2 per thousand of presumed and initially histologically diagnosed myomectomy cases turned out to be uterine sarcomas after the review of the slides. Among the 280 surgeons who participated in the survey, 30 surgeons reported 44 myomectomy cases that were initially reported as leiomyoma which after a histopathological review of the slides, due to clinical symptoms, revealed a sarcoma. Among these 30 surgeons, 19 reported that a false benign myoma histological answer happened to them once, 9 reported twice, 1 doctor reported 3 times, and one reported 4 times. The probability of a fibroid being falsely diagnosed by histopathology as a benign tumour and later revised as a sarcoma has been calculated to be 0.2 per 1000. This estimation has been calculated based on the total number of 429,777 endoscopic surgeries performed by all doctors in their lifetime, as reported in this review.

## Discussion

The total number of 221 LMS was reported among 429,777 minimally invasive surgeries (laparoscopic and hysteroscopic myomectomies and LH and LSH), performed by all doctors in their lifetime. The overall reported sarcoma risk of all types of endoscopic myoma surgeries has been estimated to be 1.5% of operations. Categorizing by type, 57 (0.06%) LMS were operated by laparoscopic myomectomy and 54 (0.07%) by hysteroscopic myomectomy, while 38 (0.13%) LMS were operated by laparoscopic subtotal hysterectomy and 72 (0.31%) by laparoscopic hysterectomy.

The incidence of uterine sarcoma in presumed fibroids has been reported to be 0.14% (1:700) with a range from 0.014 to 0.49% (1:204), which is in agreement with our overall results after MIS 0.15% [2, 8, 9, 15]. The lower prevalence of sarcoma found in LSH 0.13% is also in agreement with other studies [12, 16–18]. Studies with laparoscopic supracervical hysterectomies, sometimes without the presumption of fibroids, will result in a lower reported prevalence. A difference in prevalence between studies where fibroids intended to be morcellated and the older (pathology) studies where all uteri and fibroids served as a denominator in the prevalence rate has also been demonstrated [17]. Table 3 presents several recent published studies with a large number of patients, where 7 studies including the present survey study, report the incidence of LMS after myomectomy to be between 0.03–0.09% and one study found 1.0%. A meta-analysis, based largely on peer-reviewed articles, demonstrated a prevalence of 0.14%, a similar result to literature reviews [1, 5]. However, a meta-analysis by Pritts et al., looking at prospective trials, detected 131 articles with 29,877 patients operated for fibroids and found a sarcoma prevalence of 1:7400 (0.014%) [12]. The large prevalence difference as compared to our results and the one reported in literature may be caused by a higher number of prospective trials (50%) of all languages and a statistical correction for low volume studies. A literature review addressing this apparent discrepancy concluded that the true prevalence of uterine sarcoma in presumed fibroids is not known. There is a wide range, from 0.014 to 0.45%, produced by meta-analyses mainly based on retrospective trials. The overall risk of not previously presumed sarcomatous change in the uterus from all papers was 0.14% (1 in 700). However, there were large differences between papers with figures varying from 0.49% (1 in 204) [19] to 0.056% (1 in 1788) [20] and that by Pritts et al. 2015 was even lower at 0.014% [12, 15].

**Table 3:** Myomectomy and LMS incidence publications compared to our survey results

Author and year	Journal	Study	No pts	LMS	LMS risk %
Tanos et al. present study [16]	Gyn Surg	Survey	103,576	57	0.06
Rodriguez et al. 2016 [33]	EJOG-RB	Database analysis	10,000	13	0.13
		< 49 years after myom/my			0.0012
Bojahr et al. 2015 [20]	Gyn Surg	Retrospective	8724	6	0.07
Pritts et al. 2015 [15]	Gyn Surg	Meta-analysis retrospective studies	23,926	22	0.09
Pritts et al. 2014 [12]	JMIS	Meta-analysis prospective studies	5951	2	0.03
Brohl A et al. 2015 (ref. [34])	The Oncologist	Retrospective 75–79 years	10,120		1.0
		< 30 years			< 0.002
Wright J et al. 2015 (ref. [35])	JAMA Oncol	Retrospective 496 hospitals	3220		0.09
Yuk J et al. 2015 (ref. [36])	Annals SurgOncol	National Korean population	32,085 Lpic		0.12–0.07
			69,955 Lmy		0.1–0.05

The higher risk of sarcoma found after LH as compared to LM and HM in our series may be attributed to the older age of LH patients since myomectomy is reserved most often for younger women, who have not completed their families. An additional argument might be that usually myomas treated by hysteroscopic myomectomy are significantly smaller in size compared to those found in enlarged uteri over 14 weeks. Usually, when fibroids are large in size, hysterectomy is the preferred surgery option, as in cases of peri- and postmenopausal women. According to literature the highest (absolute) number of sarcomas is found in the fourth decade, although the incidence is still extremely low [19]. The age over 40 remains the most reliable factor for triage between high and low risk for sarcoma fibroids [8]. Data on age and prevalence does not allow the estimation of an accurate risk of sarcoma in the individual patient scheduled for fibroid surgery but they may be taken into account to define a low and intermediate risk group of patients. The large variability of LMS prevalence reported reflects the rarity of the disease, the relatively small number of articles and series of patients published, and low quality of studies. In addition, the vast majority of studies did not exclude STUMP’s until recently, counting all types of sarcomatous changes hence falsely increasing the prevalence.

On average, each surgeon is expected to see two cases of LMS per lifetime while 71% of doctors stated that they have never seen a sarcoma after any of these surgery approaches, indicating the rarity of the disease. The fact that only 12.5% (216/1728) of uterine sarcomas cases are operated laparoscopically demonstrates the surgeons’ awareness and alertness about LMS once they operate a myoma. The anticipation of a sarcoma when operating on a myoma is also reflected by the small number of doctors who reported that they have operated on a sarcoma by laparoscopy (Fig. 4).

LSH is the least performed operation, and 30% of the surgeons participated in this survey do not select this type of surgery as an option. In case of LMS after LSH, an additional operation is necessary. Since the risk of sarcoma is increased above the age of 40 probably, LSH should be reserved only for younger women [8]. When LH is performed, there is a high probability to extract the uterus vaginally without any need of power morcellation. It should be also noted that while vaginal hysterectomy allows extraction of the uterus via the vagina; however, it does not allow examination of the abdominal cavity like that in laparoscopic surgery. Hence, counselling patients with fibroids and especially young with small myoma size on the issue to choose laparoscopic surgery with its established benefits or laparotomy to escape the small LMS risks related to morcellation of the fibroid should be clear [8]. In a retrospective study by Bojahr et al. [20], reporting 10731 morcellated uteri after LSH, only 0.06% sarcoma and 0.07% endometrial carcinoma were detected. A very good prognosis in terms of survival was also found upon follow-up surgery according to the oncologic guidelines. In this ESGE survey, the overall sarcoma risk after endoscopic surgery including laparoscopic and hysteroscopic myomectomy and subtotal and total laparoscopic hysterectomy is estimated to be at 0.15% of operations as compared to 0.28% reported by FDA [1]. Our findings are in agreement with the majority of the published studies so far as demonstrated in Table 3.

The old common acceptance that rapid growth of a myoma (increasing uterine size by 6 weeks’ size within 1 year) potentially indicates a uterine sarcoma is not valid since most women with a rapidly enlarging uterus do not have a sarcoma [21–25]. In addition, a sarcoma can remain indolent for a long period of time until suddenly it becomes more aggressive causing symptoms requiring further investigation. In a prospective MRI study, which evaluated 36 women with 101 fibroids at 3-month intervals for a year, rapid growth was noted mainly in myomas that were less than 5 cm in diameter while volume of more than 30% increase in 3 months was found in 37% of the myomas [21]. In a cohort study by Parker et al. among 1332 patients operated on for presumed leiomyoma, of 371 (28%) women operated for rapid growth of the uterus, only 1 patient found to have a sarcoma. None of 198 patients who met a published definition of rapid growth had a uterine sarcoma. Authors concluded the total incidence of uterine sarcoma among patients operated on for uterine leiomyoma was extremely low (0.23%) and among patients having surgery for ‘rapidly growing’ leiomyoma (0.27%) has results that do not support the concept of increased risk of sarcoma in these women [25].

There is no imaging modality that can reliably differentiate between benign leiomyomas and uterine sarcomas before deciding to morcellate or not. A retrospective nationwide cohort study during 2000–2012 from the Cancer Registry of Norway diagnosed 212 women with uterine LMS. In 54.2% (115/212) of women suffering from LMS, a malignant diagnosis was not suspected prior to surgery. The mean age at time of diagnosis was 58.1 years and the most frequent symptom was abnormal uterine bleeding in 51.9% (110/212) [26]. Parker et al. who reviewed 26 studies of uterine sarcoma published between 1962 and 1993 reported 580 patients, and the most common presenting symptom was abnormal bleeding, followed by pain and the presence of a pelvic mass [25].

LMS and fibroids are masses formed within the uterine musculature and both often have central necrosis. Trans vaginal sonography (TVS) is the first line examination to evaluate a potential uterine pathology and especially lesions concerning the myometrium. The usually described sonographic findings suggestive of sarcoma are mixed echogenic and poor echogenic parts, central necrosis, and findings of irregular vessel distribution, low impedance to flow and high peak systolic velocity as detected by colour Doppler. However, many of these characteristics may also be found in benign leiomyomas undergoing degeneration [14]. Computed tomography neither differentiates reliably between benign and malignant uterine tumours [27].

In magnetic resonance imaging (MRI), tumour irregular contour appearance and high signal intensity as well as absence of calcifications may be all suspicious but again not a reliable indicator of uterine sarcoma [24, 28]. Two small studies using different techniques of MRI with gadolinium contrast have reported specificities of 93 to 100% and positive predictive values of 53 to 100% [29, 30]. Baral et al. [30] reviewing MRI features of uterine sarcoma concluded that a combination of criteria including biological (elevated LDH and LDH isozyme), histological (transcervical-guided biopsies and expression of low-molecular mass polypeptide 2 and Ki-67), and imaging findings (solid mass with irregular margins, hyperintensity hemorrhagic changes T1-weighted fat suppressed images low value of apparent diffusion coefficient) may raise suspicion of the possibility of a malignant tumour. They also suggested that transcervical image-guided biopsy should be considered as a useful option in case of atypical imaging findings [31]. However, more prospective randomized controlled trials needed to extract firm conclusions. Positron emission tomography/CT with fluorodeoxyglucose (FDG) also failed to distinguish between leiomyomas and uterine sarcomas. Although the FDG uptake is high in LMS and low in leiomyomas, the high uptake variability among individual tumours, makes also this test unsuitable predicting the presumed myomas with sarcomatous changes [32].

### Study limitations and potential biases

Although our survey results are consistent with other prospective and retrospective meta-analysis studies, this should be evaluated with caution. Several biases might be involved and results should be elaborated with caution. The survey study has its limitations, however, which gives the opportunity to surgeons to disclose their complications without exposing directly their names. The strict data collection followed in a prospective or cohort studies is absent. The reliability of our results depends totally on the respondents, but probably balances with the method of survey since we are dealing with a rare disease and very low LMS incidence. A questionnaire has an important recall bias and uncertainty since the participants may forget the exact number of morcellated sarcomas once they are reporting their data just by memory. The fact is that most of the surgeons and especially high activity endoscopic surgery centres have been evaluating their results after the FDA announcement. In addition, the patients’ electronic registrations and reports make easy the identification and recall of rare cases. However, the actual figures are also difficult to be collected in a prospective study of such rare cases like LMS.

Just over half of the respondents 54%, had 10 years or less experience with MIS. Given that uterine LMS is a rare finding, the estimation of the LMS incidence and the results accuracy found by our survey may carry a certain limitation since the respondents had relatively little experience. However, laparoscopic myomectomy demands high level suturing skills which is more prominent to younger laparoscopic surgeons. Also, proper teaching and guidance on laparoscopic teaching started only in the last decade. In order to bypass this potential bias, the frequency and probability of sarcoma was calculated by the number and types of operations performed in a lifetime and the total number of sarcomas diagnosed in a lifetime by each surgeon as shown in Table 2. The 8.6% response rate to ESGE survey call might also be considered another bias which, however, partially can be explained that a big group of ESGE members might not perform laparoscopic myomectomy and/or would not like to share their experience with LMS cases.

## Conclusions

Taking into consideration the limitations of a survey study, our results show that laparoscopic hysterectomy and LSH carry higher risk of LMS than laparoscopic or hysteroscopic myomectomy cases, probably due to the older age of the patients and larger fibroids. Once LH is performed, the preferable way to remove the uterus is via the vagina when the size and circumstances are suitable. Probably, LSH should be discouraged in high-risk patients because the uterus should be morcellated to remove it from the pelvic cavity. According to our study, gynaecologists practicing MIS in Europe are aware of, and cautious about, LMS and the potential of spreading sarcomatous cells after power morcellation. With reserve and including all biases mentioned above, it has also been demonstrated that myomectomy by hysteroscopy or laparoscopy may have similar risk of sarcoma about 0.07%. The survey results demonstrate the cautious surgery attitude of this specific group of gynae-endoscopist about morcellation of a LMS on a presumed myoma and a big uterus. Prospective, larger and multicentre data collection may clarify further the important issue of LMS prevalence.

## Abbreviations

ESGE: European Society of Gynaecological Endoscopy
LH: Laparoscopic hysterectomy
LMS: Leiomyosarcoma
LSH: Laparoscopic subtotal hysterectomy
